# Application of MM-PBSA Methods in Virtual Screening

**DOI:** 10.3390/molecules25081971

**Published:** 2020-04-23

**Authors:** Giulio Poli, Carlotta Granchi, Flavio Rizzolio, Tiziano Tuccinardi

**Affiliations:** 1 Department of Pharmacy, University of Pisa, 56126 Pisa, Italy; giulio.poli@unipi.it (G.P.); carlotta.granchi@unipi.it (C.G.); 2 Department of Molecular science and Nanosystems, University Ca’ Foscari of Venice, 30170 Venice, Italy; flavio.rizzolio@unive.it; 3 Pathology unit, Centro di Riferimento Oncologico di Aviano (CRO) IRCCS, 33081 Aviano, Italy

**Keywords:** virtual screening, MM-PBSA, rescoring, docking

## Abstract

Computer-aided drug design techniques are today largely applied in medicinal chemistry. In particular, receptor-based virtual screening (VS) studies, in which molecular docking represents the gold standard in silico approach, constitute a powerful strategy for identifying novel hit compounds active against the desired target receptor. Nevertheless, the need for improving the ability of docking in discriminating true active ligands from inactive compounds, thus boosting VS hit rates, is still pressing. In this context, the use of binding free energy evaluation approaches can represent a profitable tool for rescoring ligand-protein complexes predicted by docking based on more reliable estimations of ligand-protein binding affinities than those obtained with simple scoring functions. In the present review, we focused our attention on the Molecular Mechanics-Poisson Boltzman Surface Area (MM-PBSA) method for the calculation of binding free energies and its application in VS studies. We provided examples of successful applications of this method in VS campaigns and evaluation studies in which the reliability of this approach has been assessed, thus providing useful guidelines for employing this approach in VS.

## 1. Introduction

Computer-aided drug design includes a vast range of different techniques that are widely applied in medicinal chemistry, at present. The different in silico approaches are generally grouped into two main categories: ligand-based and receptor-based approaches. The former methods exploit the information about the structure and properties of known ligands active on the target of interest for the identification and design of novel compounds; the latter benefit from the knowledge of the structure of the target itself, obtained through X-ray crystallography, NMR experiments or even homology modeling, in order to search for new ligands. Undoubtedly, receptor-based approaches present serious advantages compared to ligand-based methods, since they enable to directly evaluate the potential interaction of either known or novel putative ligands into the binding site of the studied target. For this purpose, molecular docking represents the gold standard approach to predict the most favorable binding disposition of a ligand within a protein target and proved to be a fundamental tool in virtual screening (VS) studies aimed at identifying novel hit compounds [1]. However, one of the main limitations of docking is related to the estimation of ligand-protein binding affinities, which are normally calculated by applying simple scoring functions on the predicted docking poses. The scoring step in docking calculations has a double scope: the evaluation and ranking of the different binding poses predicted for a single compound, in order to identify the most reliable one (which should be the top-scored), and the comparison of the binding poses predicted for different ligands, aimed at identifying the compounds that may establish strong interactions with the protein binding site and are thus more likely to actually bind to the target [2]. Both scopes are obviously important when performing docking evaluations, but when these are applied to VS studies, in which a large number of molecules are generally screened, the ranking of compounds based on their docking scores assumes a fundamental role in order to prioritize those that have more chances to be actual ligands of the target of interest, advance them to additional evaluation steps and eventually select the most promising ones for biological experiments assessing their activity. Despite the availability of different types of scoring functions that estimate ligand binding affinities based on different methods [3,4], their ability to discriminate active ligands from inactive compounds for a certain target receptor is often not sufficiently satisfying, even because it is not possible to know a priori the screening performance of a scoring function in ranking ligands of a specific target. For this reason, different strategies have been used in the attempt to overcome these limitations, such as: *a*) a thorough preliminary evaluation of various docking and scoring methods in order to select the most suitable for the target of interest [5,6]; *b*) development of target-specific scoring functions [7,8,9]; *c*) inclusion of ligand-based and pharmacophoric elements within the docking algorithm [10,11]; *d*) combination of different rescoring methods into a consensus scoring approach [12,13,14], and even combination of different binding poses predicted for each compound by multiple docking methods in order to rank the ligands based on the number of docking procedures predicting the same pose (consensus docking) [15,16,17,18,19]. Moreover, the rapid development and improvement of machine learning and deep learning techniques has recently offered new possibilities in the field of receptor-based drug design; in fact, novel tools and strategies for the binding-affinity prediction and ranking of docked compounds based on random forest models [20], neural networks [21], and other algorithms have emerged in the last few years, often demonstrating an improved performance with respect to classic scoring functions [22]. Beyond these approaches, a profitable procedure consists in performing a more accurate estimation of ligand-protein binding affinities, calculating the free energy of binding associated to the formation of the ligand-protein complexes generated by docking. One of the most commonly used methods to perform such evaluations is represented by the Molecular Mechanics-Poisson Boltzmann Surface Area (MM-PBSA) approach [23,24], which combines energetic calculations based on molecular mechanics with free energy calculations based on implicit solvent models. Precisely, the MM-PBSA method estimates the binding free energy of a ligand-protein complex as the difference between the free energy of the complex and the free energies of the unbound components (receptor and ligand). For estimating the free energy of each component, both entropic and enthalpic terms are considered and the total binding free energy (ΔG_bind_) is calculated as a sum of the gas-phase interaction energy between ligand and protein (ΔE_MM_), the solvation energy associated to the transition from the gas-phase to the solvated state (ΔG_solv_), and the change in conformational entropy associated with ligand binding (−TΔS):

ΔG_bind_ = ΔE_MM_ + ΔG_solv_ − TΔS (1)

In the MM-PBSA method, ΔE_MM_ is calculated based on molecular mechanics and considering different components, i.e., internal energy (ΔE_int_), electrostatic interaction (ΔE_elec_), and van der Waals interaction energies (ΔE_vdW_):

ΔE_MM_ = ΔE_int_ + ΔE_elec_ + ΔE_vdW_ (2)

Even the solvation free energy ΔG_solv_ is estimated as a sum of the polar and non-polar components. The polar contribution to the solvation free energy is calculated using the Poisson-Boltzmann implicit solvent model (ΔG_PB_), while the non-polar contribution is calculated based on the solvent accessible surface area (ΔG_SA_):

ΔG_solv_ = ΔG_PB_ + ΔG_SA_ (3)

The application of the Poisson-Boltzmann implicit solvent model for the estimation of the polar solvation free energy ΔG_PB_ consists in considering the solvent as a continuum, a homogeneous medium with no explicit solvent molecules, and calculating the interactions between the solute atoms and the implicit solvent by solving the Poisson-Boltzmann equation [25]. Other implicit solvent models can be used to calculate the polar solvation free energy. The generalized Born models are often used as an alternative to the Poisson-Boltzmann model; these use approximations of the Poisson-Boltzmann equation to calculate the interactions between solute and implicit solvent, which are estimated in a less accurate but considerably faster way [26]. The MM-GBSA (Molecular Mechanics-Generalized Born Surface Area) methods apply generalized Born models for calculating binding free energies; these methods differ from MM-PBSA only in the polar solvation free energy term (ΔG_GB_ instead of ΔG_PB_), since all other energy components are calculated in the same way. Finally, the entropic term is usually estimated using the normal-mode analysis performed at the molecular mechanic level [27]. This type of analysis is, however, time-consuming; thus, the entropic contribution to the total binding free energy is often not considered, although this can have deleterious effects in some cases (*vide infra*).

Binding free energy calculations can be applied for different purposes. In association to robust docking studies and sufficiently long molecular dynamics (MD) simulations, MM-PBSA and similar approaches can be employed in thorough pose prediction studies aimed at identifying the most reliable disposition of an active compound into the binding site of its target, when no information about its bioactive conformation is known [28]. Moreover, MM-PBSA evaluations can be used for better rationalizing structure-activity relationships and selectivity profiles of ligands [29] and to derive guidelines for lead-optimization studies, even in substitution of more computationally demanding procedures, such as free-energy perturbation approaches [30,31]. In these cases, the binding free energies of the complexes are calculated from several different snapshots extracted from the whole MD trajectory, in which ligand and protein can assume different conformations, and the final binding energy value is obtained by averaging the energies estimated for the different snapshots. Alternatively, another possible approach that showed to be powerful for the prediction of reliable ligand poses and binding free energies consists in performing multiple short MD simulation replicas (10–20 replicas of 1 or few ns) for a set of different binding poses generated by docking. Binding energies are then calculated with the MM-PBSA method for all different MD replicas performed for each docking pose; the binding affinity associated to each docking pose will thus correspond to the average of the obtained values. Terayama and co-workers demonstrated that this approach is particularly effective in identifying the correct binding mode of a ligand into its corresponding protein target, despite the high computation time required. In this context, the authors applied machine learning methods called Best Arm Identification (BAI) algorithms that were able to reduce the total number of MD replicas and MM-PBSA calculations to be performed, since they could optimally control the necessary number of calculations sufficient to identify the correct pose for each ligand [32]. Although these methods allowed to remarkably decrease the required computation time, the application of such approach would still be prohibitive for large libraries of compounds as in VS studies. However, the MM-PBSA method can also be performed on MD snapshots extracted from single brief MD simulations or even simple energy minimized ligand-protein complexes. This allows to considerably accelerate the computation time required to calculate the binding energy associated to a single ligand-protein complex and, thus, to evaluate a large number of complexes in a reasonable amount of time. For this reason, the MM-PBSA method has been evaluated and applied as a rescoring protocol in VS campaigns, as a strategy to overcome common limitations of simple scoring functions of docking software and to improve the screening power and the hit rates of docking-based VS approaches for the identification of new active molecules. In this review, we focused on the role of MM-PBSA in VS. We summarized results and considerations obtained from the performance assessments of various MM-PBSA protocols, also in comparison with similar methods like the MM-GBSA, which uses the generalized Born approximation of the Poisson Boltzmann equation for the evaluation of the polar solvation energies. Moreover, we provided an overview of examples in which the MM-PBSA approach has been successfully applied in various types of VS studies.

## 2. Preliminary Considerations

The studies herein described were selected to provide the reader with a panoramic view of the evaluations and applications of the MM-PBSA methods in VS. Although, we are aware that the present review does not include a fully exhaustive report of all studies dealing with this topic ever performed, it still includes all recent performance assessments and successful application of the MM-PBSA method in VS, in which the main issues related to the calibration and use of this approach in different contexts are addressed.

In general, in the evaluations and VS studies herein described, the ligand-protein complexes subjected to energy minimizations and MD simulations, were enclosed into an explicit solvent water box, together with sodium or chloride counterions added (when necessary) to assure the neutralization of the system total charge. Nevertheless, in some evaluation studies reported here, different approaches have been used for treating the solvent in the minimization and MD calculations prior to MM-PBSA evaluations, as specified (*vide infra*). Both the explicit solvent molecules and ions are however removed in MM-PBSA calculations, which are performed considering the solvent (and also ions) implicitly, as a continuum. The use of alternative models of implicit solvent, such as the generalized Born models, affects the calculation of the polar solvation free energies, and thus the total binding free energy values, as well as the computation time required for the calculations.

An important parameter to be considered in binding free energy evaluations is the solute dielectric constant or internal dielectric constant (IDC), which also corresponds to the gas-phase state (normally equal to 1), in opposition to the dielectric constant of the solvent (normally equal to 80). Changing the value of the IDC corresponds to change the polarizability of the solute, which influences the electropolar contributions to the binding free energy. It was demonstrated that for proteins with considerably charged binding interfaces, which undergo a significant change in polarizability upon binding, the use of IDC values higher than 1, which reduces the electrostatic and polar solvation energy contributions to the overall binding free energy, allowed more reliable estimations of binding energies, especially when strong polar interactions with the corresponding ligands were observed [33]. Therefore, the choice of an IDC value appropriate for the system to be analyzed may represent an important issue to be addressed when planning to use MM-PBSA methods in VS studies. However, when MM-PBSA calculations are performed for systems containing receptors and/or ligands with total charge different from zero, achieving convergence of binding free energies can be very difficult, due to the large fluctuations that can occur during the MD simulations and due to the fact that uncertainty in the calculation of the solvation energy is proportional to the polarity of the considered molecules [34]. Moreover, it is known that a positive trivial correlation between the size of a ligand and its activity toward the target receptor may often occur in drug design. In this case the major contribution to the ligands binding affinities will be represented by the van der Waals or nonpolar terms, while the electrostatic or polar contributions may add just noise and even have a negative effect in the reliability of binding affinity predictions. In this case, an increase of the IDC value can reduce this effect and improve the reliability of the method.

In the studies herein presented, these and other issues related to the use of the MM-PBSA method in VS have been addressed. Moreover, in the present review we described various VS campaigns where the MM-PBSA method has been employed in association with molecular docking into protein X-ray structures and homology models, as well as in combination with other approaches, for the identification of novel ligands of different targets. These studies span a wide range of possible applications of MM-PBSA energy evaluations in VS, including the screening of focused libraries of drug-like compounds, molecular fragments, and peptide ligands, up to large databases of commercial compounds.

## 3. MM-PBSA Evaluation Studies

Kuhn and co-workers reported in 2005 one of the first evaluations of the MM-PBSA method as a post-docking filter in VS studies [35]. A dataset of 7076 random decoys has been incorporated with 452 compounds active against seven different targets (i.e., 128 COX-2, 55 estrogen receptor, 72 p38 MAP kinase, 36 gyrase B, 67 thrombin, 43 gelatinase A, and 51 neuramidase ligands), and the so-obtained enriched dataset has been docked in the seven different targets by using FRED with the ChemScore fitness scoring function [36]. From each target, the top-ranked 200 ligands were retained, resulting in a reduced number of actives: COX-2 (42), estrogen receptor (27), p38 MAP kinase (23), gyrase (7), thrombin (36), gelatinase A (9), neuraminidase (12). The final ligand-protein complexes, were subjected to 1000 step of energy minimization with the sander module of AMBER 6, using parm94 and GAFF (general AMBER force field) force fields for proteins and ligands, respectively; the minimized complexes were then used for MM-PBSA calculations and ranked accordingly. By analyzing the seven enrichment curves and comparing the results with the FRED/Chemscore combination, the authors highlighted an overall improvement of VS performances leading in all cases to better than random prediction accuracy and improving the rank-ordering for five out of seven targets relative to FRED/ChemScore method. Furthermore, for the p38 MAP kinase, thrombin, and neuramidase targets the authors tested the possible application of MM-PBSA to rank multiple docking poses of the same ligands. To this aim, the inclusion of the three highest scored poses, after structural clustering (rmsd = 2.0 Å), into the MM-PBSA ranking led to a higher enrichment relative to the single-pose MM-PBSA results only for p38 kinase, whereas, for both thrombin and neuraminidase, results were unchanged or even worse. The authors hypothesized that the improvement for p38 kinase could be attributed to the fact that for several molecules the correct binding modes were sampled but not scored highest and the MM-PBSA analysis increased the number of predicted correct binding modes. Differently, for neuraminidase the correct binding mode was correctly identified by docking for most of the inhibitors, and adding additional poses did not lead to improved binding energies.

In 2009, Rastelli and co-workers reported the development of an automated computational procedure called BEAR (Binding Estimation After Refinement), which had the important function of placing MM-PBSA and MM-GBSA procedures at the center of VS platforms [37]. The BEAR procedure requires a preprocessing step in which hydrogens are added to the receptor and atomic charges to the ligands whose disposition resulted from previous docking calculations. The ligand-receptor complexes are energy minimized without restraints and then 100 ps of MD simulation are carried out constraining the protein and allowing the movement of the ligand. Both minimization and MD steps are performed without explicit solvent, but using distance-dependent dielectric constant conditions. Finally, the structure of the complex is re-minimized and then scored by estimating the binding free energy applying the MM-PBSA and MM-GBSA methods. In order to test the reliability of this approach in the VS field, an enriched dataset containing 14 known active inhibitors of *Plasmodium falciparum* dihydrofolate reductase (PfDHFR), together with 1706 decoys, was created. These compounds were then docked into the target receptor with Autodock 4, processed with BEAR procedure, and the results in terms of enrichment curves were evaluated in comparison with the Autodock 4 ranking results. Compared with Autodock, BEAR yielded strikingly better enrichment of known actives using either the MM-PBSA or MM-GBSA scoring function, with MM-PBSA that resulted in the best method. Interestingly, another application of BEAR focused on diverse inhibitors of aldose reductase gave a striking agreement with their experimental activities; in particular, squared correlation coefficients of 0.80 and 0.73 between computed and experimental binding free energies were obtained using MM-PBSA and MM-GBSA, respectively, thus providing significant validation of the methodology [38].

In 2011, Pricl and co-workers tested the MM-PBSA technique for identifying new σ_1_ receptor ligands [39]. They built a homology model of the σ_1_ receptor protein that was used for docking 12 known bioactive ligands into the putative binding site of the protein with Autodock 4. Then, each ligand-receptor complex was subjected to a total of 36 ns of MD simulation with the last unconstrained 20 ns that were used for the estimation of the free energy of binding. As a result, for each ligand, the binding free energy was calculated as the average of the MM-PBSA obtained from 20,000 snapshots. The predicted binding affinities resulted in a very good agreement with the experimental ones, as this comparison resulted in a quadratic correlation coefficient R^2^ of 0.93.

**Figure 1:**
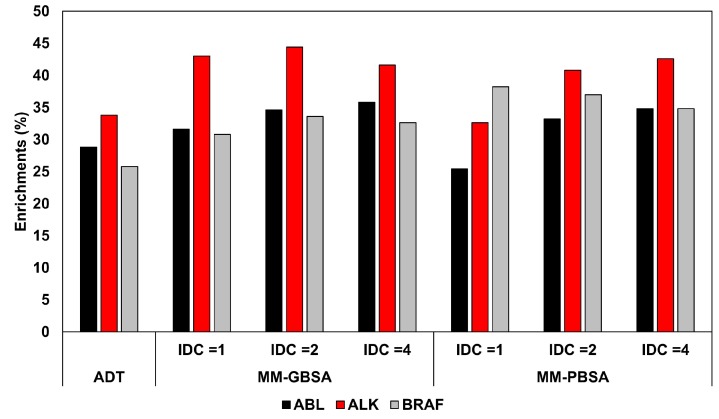
Enrichments (%) obtained for the top-scored 500 compounds of ABL, ALK, and BRAF databases, using Autodock (ADT) and the different Molecular Mechanics-Generalized Born Surface Area (MM-GBSA) and Molecular Mechanics-Poisson Boltzman Surface Area (MM-PBSA) methods tested by Hou and co-workers.

Hou and co-workers focused their attention on studying the importance of the internal dielectric constant (IDC) used in the calculation of binding free energies with MM-GBSA and MM-PBSA methods, when applied in VS protocols [40]. For this study, three tyrosine kinases were used as reference targets; in particular, three targeted enriched databases including more than 7200 compounds were created collecting 286, 342, and 402 known inhibitors of ABL, ALK, and BRAF kinases, respectively, and adding to each of them 7000 compounds selected from a commercial database based on fingerprint similarity. All compounds of each database were docked into a reference X-ray structure of the corresponding kinase using Autodock 4; subsequently, the ligand-protein complexes obtained considering the top-scored pose for each ligand were subjected to a three-stage minimization process performed with AMBER 12, using Amber03 and GAFF force fields for proteins and ligands, respectively. The first step consisted in 1000 total minimization cycles (500 using steepest descent and 500 with conjugate gradient algorithms), where the backbone heavy atoms of the protein were subjected to a harmonic restraint of 50 kcal/ Å^2^·mol; in the second step, the same 1000 minimization cycles were repeated decreasing the position restraint to 10 kcal/ Å^2^·mol; in the third and last step, 3000 minimization cycles (1000 using steepest descent and 2000 with conjugate gradient algorithms) were performed with no restraint. The energy minimized complexes were then subjected to both MM-GBSA and MM-PBSA calculations performed with AMBER 12. All energetic evaluations with both methods were repeated three times, using an IDC of 1 (default), 2 and 4 to evaluate the rescoring accuracy of a total of six different protocols. Interestingly, the results showed that the use of the default IDC generated a comparable or even worse ranking accuracy, in terms of area under the curve (AUC), with respect to Autodock, while the use of higher IDC values could provide a certain improvement of the ranking accuracy, with the best increase of AUC obtained for ABL with the MM-PBSA method (AUC = 0.898 versus 0.859). However, the improvements obtained using either MM-PBSA or MM-GBSA methods were comparable, suggesting the generalized Born approximation as the preferential choice in VS studies due to its higher calculation speed. The same analysis was then repeated considering, for each docked ligand, the three top-scored poses, thus subjecting three different ligand-protein complexes to the minimization and energy evaluation protocols. Using IDC values of 2 and 4, the results showed that the increase in screening power obtained considering more than one pose for each ligand was marginal, thus demonstrating that the use of an optimized IDC value could be also important for saving computation time. This suggests that a proper calibration of the MM-PBSA protocol is advisable in order find the best set of parameters to optimize its screening performance for a specific application. The better results obtained using IDC values higher than default were attributed to the fact that tyrosine kinases have highly charged binding sites due to few conserved charged residues. For this reason, higher IDC values may be recommended to better consider the electronic polarization effect. In order to better study the impact of the IDC in the discrimination between active and decoy compounds, the enrichment factors obtained on each database using the different protocols were calculated for the top 200, 500, and 1000 compounds. The analysis evidenced that the rescoring protocols determined remarkable improvements of the enrichments, especially relative to the to the top 500 compounds (on average, +13.1, +20.8, and +33.7% for ABL, ALK, and BRAF, respectively). However, the best results were not necessarily obtained using increased IDC values (Figure 1).

Finally, the impact of using MD simulations in combination with energy calculations was evaluated. For each database, the minimized ligand-protein complexes of each ligand, ranked based on the MM-GBSA method with IDC = 2, were considered. The top 720 complexes (approximately 10% of the database) were subjected to a total of 600 ps of MD simulation and the 50 frames corresponding to the last 500 ps were used for both MM-GBSA and MM-PBSA rescoring. The enrichments calculated for the top 200 and 500 complexes ranked according to the MD-GB/PBSA rescoring method showed only minor differences with respect to those obtained using the minimized complexes, thus suggesting that incorporating MD simulations in the rescoring protocol was unnecessary for improving the enrichment in this VS workflow. This is consistent with the high flexibility of kinases, which makes them challenging to address with MD simulations. Nevertheless, this protocol showed to improve the quality of docking results by optimizing unfavorable binding modes predicted by docking.

A similar analysis was performed by Li and Dong in 2015, studying how different EGFR kinase structures can affect the discrimination of active compounds from decoys by docking-based VS [41]. In this study an enriched database including 27 known EGFR inhibitors and the Clean Drug-Like T60 dataset, constituted by 7561 decoy compounds from ZINC database [42] was docked into 49 different EGFR structures using GLIDE standard-precision (SP) docking procedure. The discriminatory power related to each single structure was evaluated based on the enrichment factor at 1% of the screening database and then the effect of using different ensembles of protein structures was analyzed. Within this study, a subset of 17 fully solved EGFR structures (without missing protein residues or loops) and 92 compounds (among which 15 known active ligands), appearing in the top-ranked 1% of the full database when using at least 10 of these EGFR structures, were used to study the rescoring reliability of an MD/MM-PBSA method using different IDC values (1, 2, and 4). Each ligand-protein complex was subjected to energy minimization and an MD protocol, including brief unrestrained heating and equilibration steps, followed by a 10 ns production step in the NVT ensemble, which was used for energetic evaluations performed every 10 ps. The results were evaluated in terms of mean and median rank of the known active ligands. The analysis showed that using the default IDC, mean and median values around 60 were obtained, while increasing the IDC value a great improvement of rescoring reliability could be obtained. In particular, the use of IDC = 4 produced the best results, since for 15 out of the 17 EGFR structures, a median rank below or equal to 13 was obtained for the active ligands, which means that 8 out of the 15 known actives were ranked within the top 13 positions out of the total 92 compounds. The same analysis was then repeated calculating binding free energies from shorter MD time intervals (2, 4, 6, and 8 ns), thus evaluating the impact of the simulation length on the MM-PBSA rescoring power. The results showed that, using the increased IDC values, the simulation length had only negligible effects on mean and median ranking values of the known inhibitors, thus further underlying the importance of using the proper IDC values when applying MM-PBSA for VS. Nevertheless, the difference in results relative to the use of different EGFR structures was found to be reduced along with the increase of the simulation length.

In the same year, Pentikäinen tested the reliability of binding energy calculations in VS approaches focusing on 5 different targets: aldose reductase-2 (ALR-2), AmpC β-lactamase (AmpC), human heat shock protein 90 (HSP90), phosphodiesterase-5 (PDE-5), and progesterone receptor (PR) [43]. The MM-PBSA method was tested in comparison with MM-GBSA, using the three different GB models available in AMBER 10, and SIE (solvated interaction energy) method, which is still based on Poisson-Boltzman equation. For this study, five DUD [44] datasets corresponding to the five different protein targets were used: these included 26 actives and 920 decoys for ALR-2, 21 actives and 734 decoys for AmpC, 24 actives and 861 decoys for HSP90, 51 actives and 1810 decoys for PDE5, and 27 actives and 967 decoys for PR. Initially, all compounds were docked into the corresponding X-ray structure included in the DUD dataset using 10 genetic algorithm runs of the GOLD software with GoldScore fitness function. The compounds were also superimposed to the bound crystallographic ligand using SHAEP algorithm, based on shape and electrostatic similarity. For each ligand, the top-scored poses based on GoldScore fitness and SHAEP similarity score were subjected to unrestrained energy minimizations and a brief MD simulation protocol consisting in a constant volume heating step with position restraints and a constant pressure production step of 512 ps with no restraints, which was used for binding energy evaluations. In particular, 128 MD snapshots (one every 4 ps) were used for these calculations. The compounds of each dataset were ranked according to the binding energies calculated with the five different methods and using both GOLD and SHAEP poses. The results were evaluated in terms of AUC and enrichment factor at 1, 5, and 10% of the ranked database and showed to be case specific, since none of the tested procedure generally outperformed the others in all cases. In general, rescoring ligand poses according to MD-based ligand-protein binding affinities produced a clear improvement of the screening results compared to docking except for ALR-2. For AmpC and HSP90, the MM-PBSA method was found to have a lower performance with respect to the best MM-GBSA method, while it showed to perform well for the other three targets either in terms of enrichments or in terms of AUC values; however, comparable results were obtained using MM-GBSA methods. The simulation length was confirmed to have a minor impact on the VS performance of both MM-PBSA and the other methods, since repeating the analysis on the basis of shorter simulation intervals (384, 256, 128, 64, 32, and 4 ps, corresponding to 96, 64, 32, 16, 8, and 1 MD snapshots, respectively) produced similar results in terms of enrichment factors and AUC values and even single-point energy evaluations showed comparable results in many cases. Nevertheless, the increase of the MD length generated a slight reduction of AUC values for AmpC and HSP90. In the same study, the different binding free energy evaluation methods were also tested evaluating their performance in predicting binding affinities of known ligands of the selected targets retrieved from ChEMBL database [45], with known IC_50_ values. The evaluations were performed based on seven different simulation intervals illustrated above. The results showed again a pronounced case specificity, since very good correlation coefficient values (up to 0.76) using the MM-PBSA method were only obtained for ALR-2, while for all other targets, poor or even inverse correlations were observed. However, for AmC, HSP90, and PDE-5, comparable results were also produced by the other tested methods. Interestingly, what emerged from this analysis is that the reduction of the simulation interval produced an effect on the accuracy of the binding energies predicted with the MM-PBSA method, which was however target-dependent, and for most of targets shorter time intervals produced better results. Another interesting result that emerged from the whole study is that ligand-based superimposition methods might be even used as a fast alternative of docking in large VS campaigns for generating ligand-protein complexes to be analyzed through binding energy evaluations since, in some cases, the results obtained based on GOLD and SHAEP ligand poses were found to be quite similar.

A recent study performed by Contini and Hu in 2019, provided a better picture of impact and consequences on MM-PB/GBSA rescoring protocols of increasing the IDC values, revealing that this was not a universal solution for improving the performance of VS and highlighting its potential deleterious effect [46]. The analysis was carried out using three different groups of test sets: group 1 included 18 targeted databases from (Directory of Useful Decoys–Enhanced) (DUD-E) [47], including at least 100 true inactive ligands among decoys (only true active and inactive ligands were used for the study); group 2 included 19 in-house created datasets relative to 19 different targets (some of which in common to those selected from DUD-E), counting at least 200 inactive and about 10% active ligands retrieved from ChEMBL database; group 3 included the same targets of group 1, for which the 50 most active compounds of each receptor from the DUD-E were combined with 1000 decoys retrieved from the Protein Data Bank and applied in previous studies focused on the performance evaluation of docking and scoring methods in virtual screening [48,49]. For each dataset, all compounds were docked into the corresponding receptor using PLANTS, considering the top-scored pose, and the obtained ligand-protein complexes were subjected to single-frame energy evaluations with MM-PBSA method using IDC values 1, 2, 4, and 6, preceded by brief energy minimizations performed without explicit solvent, but using a generalized Born implicit solvent model. The performance of the rescoring methods was calculated based on the Receiver Operating Characteristic (ROC) AUC curves and considering not only the total binding energy but also either nonpolar or electrostatic-polar contributions. The obtained results showed that, by ranking compounds only based on the nonpolar contribution to the total binding energy, it was possible to significantly increase the VS performance with respect to docking. In particular, using PLANTS Chemplp as scoring function, a ROC AUC value higher than 0.7 was obtained for about 45% of the datasets belonging to group 1 and 2; whereas using the nonpolar energy contribution these results were obtained for about 65% of group 1 datasets and 75% of group 2 datasets. On the contrary, the ranking obtained based on the sole electrostatic-polar contribution produced ROC AUC values ≥ 0.7 for only very few (1 to 3) datasets of the same groups. Interestingly, both nonpolar and electrostatic-polar contributions were only marginally influenced by the IDC value used for energetic calculations. In fact, the values calculated with IDC 2, 4, and 6 showed very high correlation (R^2^ ≥ 0.89) with those calculated using the default IDC values. Nevertheless, the total binding free energies and the ranking of compounds based on these values still showed to be influenced by the chosen IDC (with a better performance for higher IDC values). Further analysis proved that the slight change in the electrostatic and polar energies due to the increased IDC values limited the negative effects of these energy contribution to the total binding energy, thus improving the enrichment of the total MM-PBSA estimations. This effect seemed to be primarily due to the molecular weight (MW) distribution in the dataset ligands, as observed by repeating the analyses after leveling the MW of the compounds around the range of the known active ligands. In particular, it was found that, in a MW-biased scenario, where the active compounds of the database are relatively larger than decoys and the target receptor prefers bigger ligands, the nonpolar term of the binding energy provides the main contribution to the identification of actives, while the electropolar term shows a minor role. This is a situation that often occurs in drug design; in this case, the increase of the IDC value improves the ranking performance based on the total MM-PBSA estimations reducing the electrostatic and polar contributions to the total binding free energy. On the contrary, in a MW-unbiased scenario, where actives and decoys have comparable MW, the role of the electrostatic and polar interactions well balance the nonpolar terms. Therefore, in this case, the increase of the IDC value produces a deleterious effect on the VS performance of the MM-PBSA method. Similar results were also obtained with the MM-GBSA method and using different types of ligand partial charges (AM1-BCC and Gasteiger).

## 4. MM-PBSA VS Studies

In 2007, Botta and co-workers set up a VS workflow for the identification of new RET kinase inhibitors from a focused library of synthesized compounds [50]. Starting from a homology model of the human RET kinase ATP binding domain in the closed and open state, the authors docked a focused library of 170 putative kinase inhibitors and, on the basis of their interaction with the two RET kinase models and their MM-PBSA results, ten compounds were filtered and subjected to RET inhibition assays. The resulting biological data highlighted an inhibitory activity at a concentration of 10 µM ranging from 10 to 71%, with respect to the staurosporine reference ligand.

**Figure 2:**
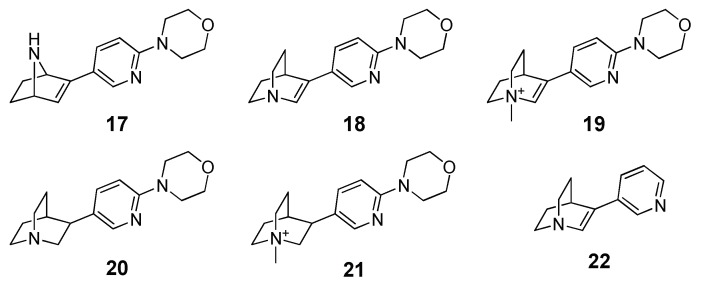
Structures of the five ligands selected through the virtual screening (VS) study (compounds **17**–**21**) performed by Grazioso and co-workers, together with the reference compound (**22**).

In 2009, Grazioso and co-workers reported a VS study aimed at the identification of new α7-subtype-preferring nicotinic acetylcholine receptor (nAChR) agonists [51]. Prior to this analysis, the authors tested the procedure by using 16 known α7 nAChR agonists characterized by a relatively wide range of affinity for the α7-subtype. These compounds were then docked into a 3D model of α7 nAChR by using Gold 3.1, and the resulting ligand-protein complexes were subjected to 500 ps of MD simulation without positional or distance constraints and then minimized. The evaluation of the binding free energy was performed by means of the MM-PBSA approach on the minimized complexes and results suggested a good correlation between the experimental and predicted binding free energy (R^2^ = 0.81, leave-one-out cross-validated q^2^ = 0.75). Furthermore, the proposed approach was undoubtedly more reliable with respect to the binding energy evaluated by using the Gold scoring function as it resulted in a R^2^ of 0.44 and a q^2^ of 0.34. Pushed by these promising results, the authors applied the herein reported procedure to predict the activity of a focused library of 150 compounds characterized by the presence of a cationic head (protonatable or permanent) linked to a substituted heteroaromatic ring with a hydrogen bond acceptor function. On the basis of the MM-PBSA ranking results, they synthesized and tested five derivatives (compounds **17**–**21**) characterized by the mutual presence of the pyridine and morpholine moieties and the reference compound 3-(pyridin-3-yl)-1-azabicylo[2.2.2]oct-2-ene (Figure 2). The resulting *K*_i_ values highlighted an activity for all the five compounds and, even though the low number of compounds invalidated the statistical significance, there was a sufficient correlation between the experimental and theoretical (R^2^ = 0.51).

In 2011, Venken and co-workers used the MM-PBSA for screening peptide derivatives active against the fusion peptide (FP) of human immunodeficiency virus type 1 (HIV-1) gp41 [52]. A 20-amino acid fragment of the protease inhibitor α-1 antitrypsin called VIRus Inhibitory Peptide (VIRIP) was already known as a molecule able to interact with the gp41 FP. Moreover, variants of this peptide with known inhibitory activity presenting mutations on specific residues were also available. The presence of a published NMR structure of one of these variants, known as VIR-165, in complex with the gp41 FP, allowed the development of a receptor-based strategy to identify novel peptide inhibitors. Starting from two VIRIP variants (VIR-165 and VIR-175), additional VIRIP derivatives were virtually designed through single or multiple mutations of the peptide sequences, thus obtaining a focused library of 121 peptides to be used in a VS based on MD simulations and MM-PBSA calculations. Initially, the protocol was calibrated using 29 reference peptides, among which VIRIP, VIR-165, VIR-175, and other active and inactive peptides, with IC_50_ values ranging from 0.18 to >100 µM. These VIRIP derivatives were subjected to a MD protocol consisting of energy minimization, an equilibration stage of 100 ps with position restraints on all the heavy atoms of the system and three different replicas of a production stage of 10 ns with different types and entities of restraints. The final 5 ns of each replica (100 snapshots, one every 10 ps) generated for each VIRIP derivative were used to predict the binding energies of the peptides and to evaluate the correlation between theoretical and experimental relative binding free energies. In these calculations, the entropic contribution to the binding free energies was also estimated with the quasi-harmonic approximation analysis performed on all 10 ns of MD. The results showed that using weak position and dihedral restraints (100 kJ/mol·nm) on the peptides backbone in MD simulations allowed to obtain a certain correlation (R^2^ = 0.40) and a good discrimination between active and inactive peptides, evaluated using ROC curves. Additionally, the analysis was repeated performing MM-PBSA calculations using different IDC values (from 2 to 6) for the different peptides depending on their amino acid composition, similarly to what performed in computational alanine scanning mutagenesis studies [53]. In practice, an IDC value of 2 was used for VIRIP peptide, as a reference; for VIRIP variants, the IDC value was increased only if the change in the amino acid sequences with respect to VIRIP determined a change in polarity. For instance, for derivatives with a single mutation with respect to VIRIP, IDC values of 4 or 3 were used if a non-polar residue replaced a charged or a polar residue in VIRIP. For derivatives with multiple mutations, the IDC value was chosen considering the contributes of all mutation. This way, IDC values ranging from 2 to 6 were used for the different peptide derivatives. By employing this approach, very promising results both in terms of correlation of relative binding energies (R^2^ = 0.71) and screening performance (ROC AUC = 0.96) were obtained, thus suggesting the suitability of this protocol in VS studies. Interestingly, it was confirmed that the inclusion of the entropic term in the binding free energy evaluations determined an appreciable improvement in the AUC and early enrichment of the ROC curve, compared to the results obtained considering only the enthalpy term. The optimized MD/MM-PBSA approach was used to predict the binding free energies of the 121 virtually designed peptides, which were compared with the binding energies of the reference VIR-165 and VIR-175 derivatives. The 20 most promising peptides were synthesized and tested for their inhibitory activity in two different types of cellular assays, using X4-tropic and R5-tropic HIV-1 virus. None of the tested peptides was found to be inactive or poorly active, as they generally showed submicromolar IC_50_ values (Table 1). Although the inhibitory activities were all comparable, some derivatives showed an appreciable potency improvement in at least one cellular assay and three VIR-165 derivatives were found to be two-fold more potent than the reference peptide against X4-tropic HIV-1.

**Figure 3:**
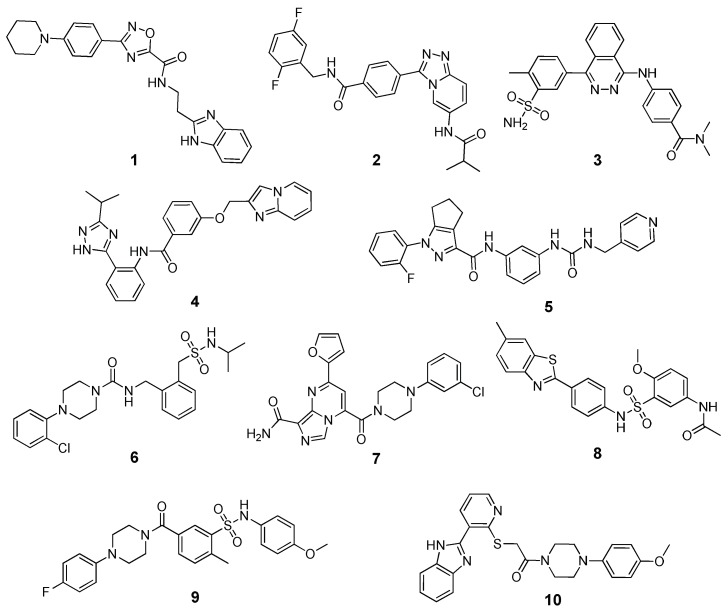
Structure of the 10 ligands selected through the VS study (compounds **1**–**10**) performed by Banoglu and co-workers.

Banoglu and co-workers recently applied binding energy evaluations with the MM-PBSA in a VS study aimed at identifying new inhibitors of microsomal prostaglandin E2 synthase-1 (mPGES-1) as potential anti-inflammatory drugs [54]. After pre-filtering the MolPort screening library based on Lipinki’s rule of 5, about 5 million compounds were docked into mPGES-1 X-ray structure using GLIDE SP and the 40,000 top-scored compounds were then docked in a different structure of the same target using the same docking procedure. The top-scored 10,000 ligands were then subjected to a diversity-based selection that allowed to obtain 1000 ligands with chemically diverse scaffolds according to MACCS (Molecular ACCess System) and pharmacophore-based fingerprints generated with Canvas. These compounds were then analyzed using the PLIP (protein-ligand interaction profiler) open source software, and 49 compounds were then selected based on their predicted interactions with the target. A final set of 10 compounds chosen after visual inspection were then subjected to a 20 ns MD simulation protocol performed with Gromacs, followed by binding energy evaluations and per-residue decomposition analysis based on the MM-PBSA method, using 50 MD snapshots extracted from the last nanosecond of simulation. The binding energy values of the selected ligands (Figure 3) were compared with those estimated for the two crystallographic ligands of the reference complexes used in the study. Interestingly, two of the 10 selected ligands (compounds **6** and **8**), which were all purchased and subjected to biological assays, showed inhibitory activities in the low micromolar range (IC_50_ = 1.2–1.3 µM) and were predicted to have higher binding energies than those of the reference inhibitors. A preliminary structural optimization of compound **8** allowed the identification of three derivatives with submicromolar activities (IC_50_ values between 0.3 and 0.6 µM).

**Figure 4:**
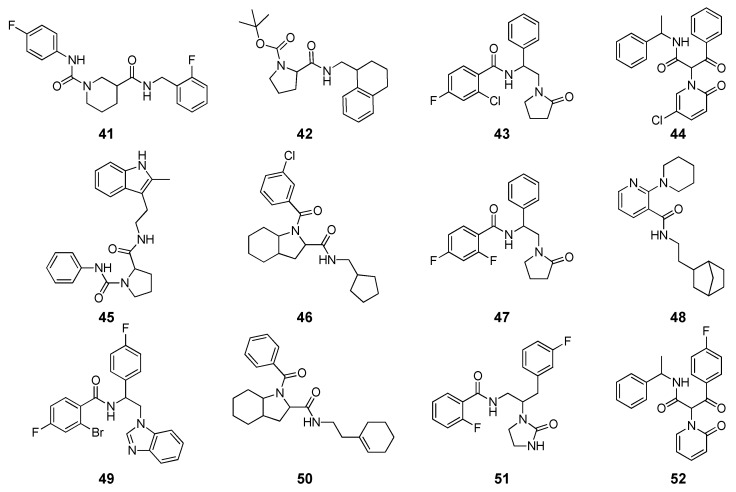
Structure of the 12 ligands selected through the VS study (compounds **41**–**52**) performed by Nakagawa and co-workers.

Nakagawa and co-workers performed a receptor-based VS study to identify novel inhibitors of the ecdysone receptor (EcR) as attractive candidates for new insecticides [55]. For this aim, a homology model of EcR was generated, and 40 reference EcR inhibitors belonging to four different structural classes of compounds were used to test the reliability of docking and binding energy methods. The ligands were first docked within the receptor binding site using FRED software with Chemgauss4 scoring function, and then three different types of energy evaluations were performed with the MM-PBSA module of AMBER16, to study their correlation with the activity of the reference compounds expressed as pIC_50_. The simplest procedure consisted in a single-point binding energy evaluations; then the ligand-protein complexes were subjected to an MD simulation protocol including 200 ps of system heating/equilibration and 150 ps of unrestrained constant pressure MD. The production step was performed in three different replicas per ligand, and the total 450 MD snapshots obtained were used for MM-PBSA calculations. Finally, the ligand conformational free energy of each compound, including a fast estimation of the ligand conformational entropy, was calculated using the FREEFORM module of SZYBKI, based on 45 out of the 450 MD snapshots and combined with the binding energy calculated with the MD-based protocol. The results showed that the docking score poorly correlated with the activity of the reference compounds (R^2^ = 0.21); interestingly, while the single-point binding energies showed higher correlation (R^2^ = 0.38), the MD-based protocol generated results even worse than those obtained with docking (R^2^ = 0.18). Nevertheless, by combining ligand conformational and binding energies derived from the MD, a significantly improved correlation was obtained (R^2^ = 0.56). Based on these data, a hierarchical receptor-based VS approach was applied on a database counting more than 3.8 million commercial compounds. First, all compounds were docked into the EcR homology model with FRED, and the top-scored 5000 molecules were subjected to single-point MM-PBSA rescoring. Then, 389 compounds with binding energy lower than −10 kcal/mol, were subjected to the above mentioned MD/MM-PBSA protocol coupled with ligand conformational energy calculations. Among ligands with ΔG of binding ≤ −9 kcal/mol, 12 compounds (Figure 4) were selected based on their structural novelty and commercial availability, after visual inspection. Binding assays revealed an inhibitory activity in the micromolar range for 5 of the tested ligands (Table 2). The proline-based compound **42**, with an IC_50_ of 9.1 µM, was the most active ligand and was considered as a starting point for future structural optimization.

Finally, a fragment-based screening study involving the use of binding energy evaluations was performed by Johnson and Hevener in 2013, which tested a docking and MD/MM-PBSA protocol for fragment-based drug discovery, aimed at identifying novel molecular fragments inhibiting N5-CAIR mutase (PurE) [56]. In this study, an in-house fragment library of 352 unique compounds with MW < 300 Da (384 fragments, considering also alternative tautomers and stereoisomers) were docked within the PurE binding site employing 100 runs of GOLD genetic algorithm with GoldScore fitness function, using the “Generate Diverse Solution” option, thus producing 100 different docking poses per molecule. For each fragment, the top-scored pose and the most different pose from this latter in terms of root-mean-square deviation (RMSD) were both subjected to energy minimization and an MD simulation protocol, including 600 ps of heating/equilibration stage with decreasing position restraints and 8 ns of production run without restraints. Subsequently, 100 MD snapshots from the last 2 ns of simulation (one every 20 ps) were used to calculate the binding energy of the fragment; in this case, the entropy term was included in the calculation using the normal-mode analysis based on 12 regularly spaced snapshots among the last 2 ns of simulation. Only the best binding energy among those estimated for all conformations/tautomers/stereoisomers of each compound was eventually considered and used to calculate its ligand efficiency (LE, defined as the ratio between the calculated ΔG of binding and the number of heavy atoms). Compounds with LE > 0.3 were considered as VS hits, and, by using this protocol, 103 VS hits were identified. Interestingly, 83 out of the 103 hits were confirmed as PurE binders by either NMR or SPR (Surface Plasmon Resonance) techniques; moreover, the MD/MM-PBSA protocol showed a significantly higher performance in ranking the true binders (based on ΔG values and corresponding LE values) than simple docking score (and heavy atoms normalized score), in terms of AUC ROC, enrichment factor, and true positive rate. However, competition binding experiments performed using STD-NMR on 65 experimental hits determined using NMR showed that only 20 fragments were actually binding to the catalytic site of the enzymes, while the others were nonspecific binders. Nevertheless, 16 out of these 20 fragments were identified by the MD/MM-PBSA protocol. Moreover, considering only the 65 compounds subjected to STD-NMR experiment as total fragment library and the 20 competitive binders as true active ligands, the MD/MM-PBSA protocol still showed better performance in compound ranking with respect to the docking score. Interestingly, it was found that by ranking the competing fragments based on the sole MM-PBSA enthalpy term, an AUC ROC of 0.76 was obtained and including also the entropy term this value underwent only a slight increase (AUC ROC = 0.79); however, the early enrichment of the curve showed a remarkable improvement, which may justify the computation time required for entropy calculations. Further analyses aimed at better investigating the different variables affecting the reliability of the protocol showed results consistent with previous evaluation studies. An IDC value of 2 produced better results in terms of enrichments and AUC ROC values with respect to IDC = 1 or 4; this is consistent with the presence of two charged residues in the active site of the enzyme that could form strong polar interactions with the fragments and may thus justify the use of an IDC value higher than default. Differently, the length of the MD simulation used for binding energy predictions (either 2, 4, or 8 ns) had only a slight influence on the calculated energy values. Finally, the use of multiple conformations per ligand improved the performance of the protocol compared with the use of a single pose; this could be due to the fact that molecular fragments, having lower size and forming weaker overall interactions with respect to drug-like ligands, may interact with the binding site in different ways. Therefore, the use of multiple binding poses would be recommended when applying MM-PBSA calculations in fragment-based screenings.

## 5. Conclusions

In the present review, we summarized the results of recent studies focused on the reliability analysis of ligand-protein binding energy evaluations based on the MM-PBSA method in VS. We provided examples of various VS studies in which this procedure has been profitably applied for the identification of novel ligands of different targets. What emerges from the different studies reviewed is that the use of binding free energy calculations for ranking and prioritizing compounds in VS studies can surely represent an effective approach that can statistically improve the screening performance with respect to simple docking studies. However, it seems that the benefits obtained by using this type of approach are rather influenced by both the target on which the VS is focused on and the virtual library of compounds to be screened. In particular, it seems that the features of the receptor binding site, and also the properties of the screened compounds, should be considered in the attempt to use the most appropriate protocol for performing energy evaluations for VS purposes. For this reason, a preliminary reliability assessment and case specific calibration of the protocol should be performed in order to obtain the best results from this approach in relation to the computation time required. Nevertheless, it seems that, especially when large compound databases are screened, energy evaluations can be safely performed on protein-ligand complexes subjected to simple energy minimizations or short MD simulations, as longer simulation times seem not to significantly improve the VS performance. Based on these considerations, large evaluation studies focused on a vast range of different protein targets would be desirable in order to better assess the domain of applicability of the MM-PBSA method in VS and to derive some guidelines relative to the best settings that should be used according to the type of target of interest. Moreover, although the performance of the MM-PBSA method has been compared to similar binding energy evaluation approaches, a large reliability assessment in comparison with different techniques, such as common scoring functions used in docking algorithms or even consensus scoring approaches, as well as recently emerging machine learning-based rescoring methods, is still missing. It would be necessary to understand if even less time consuming rescoring techniques could generate either similar or even better performance improvements. Finally, it should be considered that, due to the high computation time required, the entropic term of the binding energy is often not considered for MM-PBSA calculations and related approaches both in evaluation and application studies. However, when this term was included in the calculations, even with alternative approaches, it showed to provide an improvement of results. Therefore, we believe that the importance of the entropic term in binding free energy calculations should be evaluated in depth in order to assess its qualitative and quantitative impact on energy estimations and compound ranking, as well as to possibly identify some faster strategies able to either take into account entropy or compensate for its absence in the case that entropy is not considered.

## Figures and Tables

**Table 1:** Predicted binding free energies (kcal/mol) and experimental IC_50_ values (µM) for VIR-165, VIR-175 and their variants selected through the virtual screening (VS) study of Venken and co-workers. IC_50_ values obtained using X4-tropic and R5-tropic human immunodeficiency virus type 1 (HIV-1) virus are reported as IC_50_ X4 and IC_50_ R5, respectively.

VIR Peptide Variant	ΔG_bind_	IC_50_ X4	IC_50_ R5
VIR-165 (reference)	−36.92	0.34	0.59
VIR-175 (reference)	−32.66	0.47	0.20
VIR-165_V19T	−41.35	0.83	0.51
VIR-165_I8Q	−41.02	1.03	0.80
VIR-165_I4Y	−39.52	1.10	0.30
VIR-165_I4L	−39.43	0.62	2.03
VIR-165_I8L	−38.8	0.35	0.83
VIR-165_A13F	−38.19	0.22	0.46
VIR-165_F18L	−37.88	0.48	0.40
VIR-165_F14W	−37.28	0.33	2.51
VIR-165_I4F	−37.21	0.40	0.26
VIR-165_F18Y	−37.17	0.62	0.48
VIR-165_F12W	−37.08	0.18	1.95
VIR-165_S7Q	−36.98	0.15	0.51
VIR-165_F12L_A13F	−35.76	0.13	0.41
VIR-165_F12V_A13F_P10A	−40.73	0.61	n.d. ^a^
VIR-165_F12V_A13F_F20Y	−36.28	0.72	1.29
VIR-165_I8Y_F12V_A13F	−36.06	0.25	0.48
VIR-165_S7N_F12V_A13F	−35.18	0.24	0.21
VIR-175_I4Q	−36.35	1.61	0.62
VIR-175_M6L	−35.12	0.37	0.20
VIR-175_E2Q	−34.74	0.77	0.69

^a^ Not determined.

**Table 2:** Calculated binding free energies (kcal/mol) and experimental activities of the compound selected through the VS protocol performed by Nakagawa and co-workers.

Compound N.	ΔG_bind_	pIC_50_
**41**	−9.13	4.28
**42**	−9.49	5.04
**43**	−14.01	< 3.60
**44**	−9.57	< 4.00
**45**	−9.44	< 4.00
**46**	−10.73	4.13
**47**	−12.99	< 3.60
**48**	−9.61	4.11
**49**	−10.70	< 3.60
**50**	−11.01	4.12
**51**	−10.16	< 3.60
**52**	−10.51	< 3.60
